# Unbiased chemokine receptor screening reveals similar efficacy of lymph node- and tumor-targeted T cell immunotherapy

**DOI:** 10.1007/s00262-023-03472-w

**Published:** 2023-06-10

**Authors:** Ludwig O. Pachmayr, Anton Muehlbauer, Sophie Flommersfeld, Franziska Graml, Julian Hoenninger, Louisa von Baumgarten, Veit R. Buchholz, Simon Grassmann

**Affiliations:** 1 Institute for Medical Microbiology, Immunology and Hygiene, School of Medicine, Technical University of Munich (TUM), Munich, Germany; 2 Department of Neurosurgery, Ludwigs-Maximilians-University (LMU), Munich, Germany; 3 Present Address: Immunology Program, Memorial Sloan Kettering Cancer Center, New York, NY USA

**Keywords:** Adoptive T cell therapy, Chemokine receptors, Tumor homing, Solid tumors, Cancer immunotherapy

## Abstract

Localization is a crucial prerequisite for immune cell function and solid tumors evade immune control by modulating immune cell infiltration into the tumor stroma. Immunosuppressive cells like regulatory T cells are attracted, while cytotoxic CD8^+^ T cells are excluded. Engineering CD8^+^ T cells with chemokine receptors is a potent strategy to turn this mechanism of directed immune cell recruitment against the tumor. Here, we utilized fluorescent tagging to track the migratory behavior of tumor-specific T cells engineered with a library of all murine chemokine receptors in vivo. We then asked whether chemokine receptor-mediated redirection of antigen-specific T cells into tumors or tumor-draining lymph nodes showed superior anti-tumoral activity. We found that both targeting approaches showed higher therapeutic efficacy than control T cells. However, multiple receptors conveying the same homing pattern did not augment infiltration. Instead, in the MC38 colon carcinoma model, anti-tumoral efficacy as well as lymph node vs. tumor-homing patterns were mostly driven by CCR4 and CCR6, respectively. Overall, our data, based on fluorescent receptor tagging, identify the tumor-draining lymph node and the tumor itself as viable targets for chemokine receptor-mediated enhancement of adoptive T cell therapy.

**Supplementary Information:**

The online version contains supplementary material available at 10.1007/s00262-023-03472-w.

## Introduction

Traditionally, CD8^+^ T cells are considered to require three signals for optimal functionality: engagement of the antigen receptor, co-stimulation and stimulation by cytokines. However, none of these signals induce protective immunity without correct localization of the T cell. ‘Signal 0’ for a T cell is therefore its active migration toward a priming location or target organ and its interacting cell partners such as antigen-presenting or target cells.

Solid tumors capitalize on the location dependency of anti-tumoral immune cell function by controlling the composition of the intra-tumoral milieu. Through immunoediting, tumors can evolve to secrete a chemokine profile that attracts immunosuppressive cells such as Treg [1–3] and immunosuppressive myeloid cells [3–5]. Conversely, chemokines that facilitate infiltration of anti-tumoral immune cells such as CD8^+^ T cells and NK cells are suppressed. A crucial chemokine receptor that theoretically can recruit anti-tumor immune cells into tumors is CXCR3 [6, 7]. If solid tumors fail to suppress secretion of ligands for CXCR3 such as CXCL9 and CXCL10, this renders such tumors susceptible to immune checkpoint blockade [8]. If tumors successfully shut down expression of such anti-tumoral chemokines, immune cells are deprived of a path into the tumor.

Genetic engineering of T cells used for adoptive T cell therapy can overcome the crucial bottleneck of impaired tumor infiltration and even turn the tumor’s weapons against itself. Expression of chemokine receptors that match ligands secreted by the tumor such as CCL2, CCL22, CXCL16, CX3CL1, CXCL1 and CXCL8 can enable CD8^+^ T cells to enter the tumor [9–13]. Importantly, these chemokines are secreted by the tumor for a reason, namely because they are pro-tumorigenic [14]. Chemokines such as CCL2 and CXCL16 can have direct effects on tumor cells, promoting cell motility and invasion [15, 16]. CCL2 and CXCL8 can promote angiogenesis by acting on intra-tumoral endothelial cells [17, 18]. Furthermore, immunosuppressive cells are recruited into the tumor. CCL2 for example facilitates infiltration by immunosuppressive myeloid cells [3], whereas chemokines such as CCL22 promote recruitment of Treg cells [19]. Expression of the corresponding chemokine receptors in CD8^+^ T cells is particularly attractive because the tumor can only shut down expression of these chemokines at the cost of losing their tumorigenic function [13].

Despite the potential of approaches to genetically engineer T cells to home into the tumor, so far none has been approved for therapy in humans. A possible explanation for this is that one chemokine receptor alone is often not sufficient to induce homing into the tumor site. Another explanation could be that strategies that attempt to drive T cells directly into the tumor are sub-optimal. Since stem-like CD8^+^ T cells have recently been found to reside mostly in tumor-draining lymph nodes [20], it could be important to engineer T cells to promote homing into draining lymph nodes instead.

To answer such questions in an unbiased manner, we generated a library of all known murine chemokine receptors outfitted with one of four distinct fluorescent tags. After transduction of this fluorescently-tagged receptor library into tumor antigen-specific T cells, we assessed the homing pattern conveyed by each chemokine receptor. Next, we defined batches of tumor-homing receptors and receptors that directed T cells into tumor-draining lymph nodes and investigated if combinatorial transduction [21–23] with multiple chemokine receptors improved homing to the target organ. To our surprise, we found that while both tumor- and lymph node-homing batches enhanced tumor regression in the MC38 colon carcinoma model, transduction with multiple receptors did not enhance target organ infiltration. Instead, organ infiltration and therapeutic efficacy were largely dependent on transduction with CCR4 in the lymph node-homing batch and CCR6 in the tumor-homing batch. Interestingly, combination of these two receptors appeared to reduce treatment efficacy. Taken together, CCR4 and CCR6 provided T cells with a unique but likely not additive quality that rendered them superior over other chemokine receptors with similar homing characteristics.

## Materials and methods

### Mice

Female C57BL/JOlaHSd of 6–14 weeks were purchased from Envigo. 8–20 weeks old SIINFEKL peptide-specific TCR-transgenic OT-1 mice (C57BL/6-Tg(TcraTcrb)1100Mjb/J) were originally obtained from The Jackson Laboratory and bred under specific pathogen-free conditions at our mouse facility at the Technical University of Munich. All animal experiments were approved by local authorities and performed in accordance with national guidelines.

### Cell lines

The Platinum-E packaging cell line, OVA-expressing MC38 (MC38-OVA) cells and OVA-expressing Panc02 cells were grown in cDMEM (DMEM (Life Technologies) supplemented with 10% fetal calf serum (FCS, Sigma Aldrich), 0.025% l-Glutamine (Sigma Aldrich), 0.1% HEPES (Carl Roth), 0.001% gentamycin (Life Technologies) and 0.002% streptomycin (Life Technologies)). Primary murine T cells were cultured in cRPMI (RPMI 1640 (Life Technologies) supplemented with 10% fetal calf serum (FCS, Sigma Aldrich), 0.025% l-Glutamine (Sigma Aldrich), 0.1% HEPES (Carl Roth), 0.001% gentamycin (Life Technologies) and 0.002% streptomycin (Life Technologies)).

### RNA isolation and murine chemokine receptor cDNA amplification

Single cell suspensions from C57BL/JOlaHSd tissue samples (spleen, lymph node, Peyer’s plaque) were resuspended in 1 mL TRI Reagent (Sigma Aldrich, Cat# 93289), briefly incubated and 200 µL Chloroform (Carl Roth, Cat# 3313) was added. Phase separation was performed at 12,000 g for 15 min at 4 °C in a tabletop centrifuge. The RNA precipitation was achieved by addition of 500 µL Isopropanol (Carl Roth, Cat# 9781) to the upper layer after phase separation. The RNA pellet was washed with 1 mL 70% ice cold Ethanol. Reverse transcription was performed with 5 µg of the isolated total RNA using the AffinityScript cDNA Synthesis Kit (Agilent, Cat# 200436).

### Generation of retroviral fluorescently labeled murine chemokine receptor vectors

All fusion constructs of murine chemokine receptors and fluorescent proteins (FPs) were generated by overlap extension PCR and cloned into the retroviral pMP71 vector (a kind gift from Wolfgang Uckert) for recombinant expression in target cells. For retrovirus production, Platinum-E packaging cells were transfected via calcium phosphate precipitation with retroviral vectors encoding for all known signaling murine chemokine receptors tagged by an individual fluorescent protein (GFP, YFP, T-Sapphire or BFP) via a translational skip motif (P2A). Virus-containing supernatants were collected 48 h after transfection and purified from remaining cells by centrifugation (1500 rpm, 4 °C, 7 min). Supernatants were stored at 4 °C and used within 4 weeks after harvest.

### Generation of chemokine receptor-engineered OT-1 T cells

Spleens of OT-1 TCR-transgenic mice were harvested and brought into single-cell suspension by mashing through a 40 µm cell strainer, followed by red blood cell lysis. The prepared lymphocytes were seeded into 6-well plates (Corning) at a density of 2 × 10^6^ cells/mL in cRPMI, 0.5 µg/ml anti-CD3 antibody (Biolegend), 0.1 µg/ml anti-CD28 antibody (Biolegend) and 25 IU/mL IL-2 (Peprotech). Cells were kept in stimulation medium for 24 h. Retroviral transduction was performed in 24-well tissue culture untreated (Costar) plates pre-coated with 250 µl PBS containing 0.5 µg/ml RetroNectin® (TaKaRa Biotech), 0.5 µg/ml anti-CD3 antibody (Biolegend) and 0.1 µg/ml anti-CD28 antibody (Biolegend). Diluted virus-containing Platinum-E supernatant was spun down at 3000 g for 2 h, either at one construct per well (single transduction) or multiple constructs per well (combinatorial transduction). Supernatants were carefully discarded before adding 5 × 10^5^ activated OT-1 T cells in cRPMI supplemented with 25 IU/mL IL-2 (Peprotech), followed by centrifugation at 800 g for 1.5 h. Transduced cells were harvested after 48 h and subjected to flow cytometric analysis or adoptive transfer.

### Subcutaneous inoculation of syngeneic tumors and growth monitoring

MC38-OVA and Panc-OVA cells were grown to a confluence of 90%. Medium was removed and cells were rinsed once with PBS prior to enzymatic detachment with trypsin. Digestion was stopped by addition of fresh cDMEM and cells were pelleted. Tumor cells were resuspended in an adequate volume of PBS and cell numbers were determined by trypan-blue staining and counting. Cell numbers were adjusted to 0.5–1 × 10^6^ cells/100 µL. 100 µL of cell suspension was injected in the right flank of isofluorane anaesthetized C57BL/6JOlaHsd mice which had been subjected to total body irradiation (TBI) at 4.5 Gy 24 h prior to tumor implantation. Tumor sizes, defined as tumor area (length × width), were measured every 2–4 days using a digital caliper.

### Cell sorting and adoptive transfer of chemokine receptor-engineered OT-1 T cells

48 h after transduction, cells were stained with respective antibodies for 30 min at 4 °C in the dark, propidium iodide was used for live/dead discrimination. For survival experiments, equal numbers of CD8^+^ FP^+^ (FPs corresponding to chemokine receptor expression) PI^−^ T cells were sorted. Sorting of chemokine receptor-engineered T cells was performed on a BD FACSAria III (Becton Dickinson) or MoFlo Astrios cell sorter (Beckman Coulter). T cells were sorted into tubes pre-loaded with FCS and cell numbers were adjusted. The resulting infusion product was injected i.p. into tumor-bearing C57BL/6JOlaHsd mice. For infiltration and phenotypic analyses, transduced T cells were not subjected to cell sorting but analyzed by flow cytometry to determine initial ratios of single or combinatorial chemokine receptor-engineered T cells.

For assessment of homing preferences, the number of T cells transduced with a fluorescently-tagged chemokine receptor was normalized to the number of untransduced T cells and then compared between the organ infiltrate (at day 4 after injection) and the infusion product (at day 0 before injection). If, e.g., 50 out of 500 cells (50/500 = 0.1), sampled from the infusion product, were CCR6 positive (day 0 before injection) and 300 out of 1000 transferred T cells (300/1000 = 0.3) were later found to be CCR6 positive in the tumor (day 4 after injection), the ratio day 4/day 0 would be 0.3/0.1 = 3.

Different groups received the same number of OT-1 T cells within each experiment. For experiments shown in Fig. 3A and B we transferred on average 400.000 cells, for Fig. 4E 100.000 cells and for Fig. 4C and F 50.000 cells/mouse. For experiments shown in Fig. 3A and B, 69.1% (SEM 25.9, tumor-homing batch) and 77.4% (SEM 17.57, lymph node-homing batch) of transferred cells expressed the transduced constructs, respectively. Experiments in Fig. 4C–F were performed with T cells sorted via flow cytometry to a level of construct positivity > 95% using a BD FACSAria III (Becton Dickinson) or MoFlo Astrios cell sorter (Beckman Coulter).

### Flow cytometry

Spleens and lymph nodes were harvested and mashed through a 40 µm cell strainer to generate a single-cell suspension. Tumors were minced by scissors and digested in 1 mL RPMI 1640 supplemented with 1 mg/mL collagenase IV and 100 µg/mL DNase I (Sigma-Aldrich, respectively) for 1 h at 37 °C in a table top incubator. The tumor suspension was passed through a 100 µM mesh and rinsed with 10 mL PBS before pelleting and resuspending in 3 mL of 40% Percoll (Sigma-Aldrich) solution. Separation of the lymphocyte fraction was performed by centrifugation at 2600 rpm at room temperature for 20 min (acceleration 5 of 9, deceleration 0 of 9). After red blood cell lysis, cells from each tissue were incubated with mouse anti-CD16/32 for 15 min at 4 °C. Cells were washed with FACS buffer (PBS with 0.5% BSA and 2 mM EDTA) and stained with respective fluorophore-conjugated antibodies for 30 min at 4 °C in the dark. After washing, cells were resuspended in FACS buffer containing 1% paraformaldehyde and analyzed by flow cytometry (Cytoflex S or LX, Beckman Coulter). FlowJo software (FlowJo LLC) was used for analysis. Normalized mean fluorescent intensities (MFIs) were calculated by dividing the MFI of transduced cells by the MFI of co-transferred, untransduced cells in the respective organ.

### Statistical analyses

Significances were calculated after testing for normal distribution using unpaired *t*-test. Significance of survival differences was calculated using Log-rank test.

#### Key resources table

**Table:** 

Reagent or resource	Source	Identifier
*Antibodies*		
Anti-mouse CD8	BioLegend	53–6.7
Anti-mouse CD45.1	BioLegend	A20
Anti-mouse CD45.1	Becton Dickinson	A20
Anti-mouse CD45.2	BioLegend	104
Anti-mouse CD62L	BioLegend	MEL-14
Anti-mouse CD62L	Becton Dickinson	MEL-14
Anti-mouse PD-1	BioLegend	29F.1A12
Anti-mouse TIM-3	BioLegend	RMT3-23
Anti-mouse Ly108	BioLegend	330-AJ
Anti-mouse CCR4	BioLegend	M1/70
Anti-mouse CCR7	BioLegend	2F1
Anti-mouse CCR6	BioLegend	GB11
Anti-mouse CXCR1	BioLegend	145-2C11
Anti-mouse CD3e	BioLegend	145-2C11
Anti-mouse CD19	Becton Dickinson	1D3
Anti-mouse CD19	Becton Dickinson	HIB19
*cDNA synthesis kit*		
AffinityScript cDNA Synthesis Kit	Agilent	Cat# 200,436
*Chemicals and recombinant proteins*		
Propidium iodide (PI)	Life technologies	Cat# P1304MP
Recombinant murine IL-2	PeproTech	Cat# 213–13
RetroNectin®	Takara bio Europe	Cat# T100B
Percoll	Sigma-Aldrich	Cat# GE17-0891–01
Collagenase IV	Sigma-Aldrich	Cat# C2139
DNase I	Sigma-Aldrich	Cat# D4513-1VL
TRI Reagent®	Sigma-Aldrich	Cat# 93,289
Chloroform	Carl Roth	Cat# 3313
2-Propanol	Carl Roth	Cat# 9781
*Experimental models: cell lines*		
MC38-OVA	Bavarian Nordic	N/A
Panc-OVA	MIH	N/A
Platinum E cells	Cell Biolabs	RV-101
*Experimental models: organisms/strains*		
C57BL/6-Tg(TcraTcrb)1100Mjb/J	The Jackson Laboratory	003831
B6.SJL-Ptprca Pepcb/BoyJ	The Jackson Laboratory	002014
B6.PL-Thy1a/CyJ	The Jackson Laboratory	000406
C57BL/6JOlaHsd	Envigo	N/A
*Oligonucleotides*		
GFP family NotI fwd 5′ATTAGCGGCCGCGCCACCATGGTGAGCAAGGGCG 3′	Sigma Aldrich	N/A
GFP family EcoR1 rev 5′TAATGAATTCTTACTTGTACAGCTCG 3′	Sigma Aldrich	N/A
mCCR1 NotI fwd 5′ATTCGCGGCCGCGCCACCATGGAGATTTCAGATTTG 3′	Sigma Aldrich	N/A
mCCR1 BamHI p2a rev 5′TAATGGATCCGAAGCGAGCAGAGAGCTCATG 3′	Sigma Aldrich	N/A
mCCR2 NotI fwd 5′ATTAGCGGCCGCGCCACCATGGTGAGCAAGGGCG 3′	Sigma Aldrich	N/A
mCCR2 BamHI p2a rev 5′ATTAGCGGCCGCGCCACCATGGTGAGCAAGGGCG 3′	Sigma Aldrich	N/A
mCCR3 NotI fwd 5′TTAGCGGCCGCGCCACCATGGCATTCAACACAGATG 3′	Sigma Aldrich	N/A
mCCR3 BamHI p2a rev 5′TAATGGATCCAAACACCACAGAGCGTTG 3′	Sigma Aldrich	N/A
mCCR4 NotI fwd 5′ATTAGCGGCCGCGCCACCATGAATCCGAGGAGGTC 3′	Sigma Aldrich	N/A
mCCR4 BamHI p2a rev 5′TAATGGATCCCAAAGCGTCACGGAAGTCATG 3′	Sigma Aldrich	N/A
mCCR5 NotI fwd 5′TAATGGATCCTAAACCAGTAGAAACTTCATG 3′	Sigma Aldrich	N/A
mCCR5 BamHI p2a rev 5′GTTCGTGGCTCCGGATCCTAAACCAGTAGAAACTTC 3′	Sigma Aldrich	N/A
mCCR6 NotI fwd 5′ATTAGCGGCCGCGCCACCATGAATTCCACAGAGTC 3′	Sigma Aldrich	N/A
mCCR6 BamHI p2a rev 5′GTTCGTGGCTCCGGATCCCATGGTAAAGGACGATGC 3′	Sigma Aldrich	N/A
mCCR7 NotI fwd 5′ ATTAGCGGCCGCGCCACCATGGACCCAG GGAAACCC 3′	Sigma Aldrich	N/A
mCCR7 BamHI p2a rev 5′ TAATGGATCCCGGGGAGAAGGTTGTGGT GG 3′	Sigma Aldrich	N/A
mCCR8 NotI fwd 5′ATTAGCGGCCGCGCCACCATGGATTACACGATGGAG 3′	Sigma Aldrich	N/A
mCCR8 BamHI p2a rev 5′TAATGGATCCCAAGATGTCATCCAGGGTGG 3′	Sigma Aldrich	N/A
mCCR9 NotI fwd 5´ATTAGCGGCCGCGCCACCATGATGCCCACAGAACTC 3´	Sigma Aldrich	N/A
mCCR9 BamHI p2a rev 5′TAATGGATCCTAGGGAGAGAGCCCCCGAAG 3′	Sigma Aldrich	N/A
mCCR10 NotI fwd 5′ATTAGCGGCCGCGCCACCATGGGGACCAAGCCCAGAG 3′	Sigma Aldrich	N/A
mCCR10 BamHI p2a rev 5′TAATGGATCCGTTGTCCCAAGAGAGACTGTG 3′	Sigma Aldrich	N/A
mXCR1NotI fwd 5′ATTAGCGGCCGCGCCACCATGGACTCAGAGTCAGATG 3′	Sigma Aldrich	N/A
mXCR1 BamHI p2a rev 5′TAATGGATCCGTAGAAGGAGGGTCCCTCATATG 3′	Sigma Aldrich	N/A
mCXCR1 HindIII fwd 5′ATTAAAAGCTTGCCACCATGGCCGAGGCTGACTAT 3′	Sigma Aldrich	N/A
mCXCR1 BamHI p2a rev 5′GTTCGTGGCTCCGGATCCATAAATAGGGGTGAGAG 3′	Sigma Aldrich	N/A
mCXCR2 NotI fwd 5′ATTAAAGCTTGCCACCATGGGAGAATTCAAGGTG 3′	Sigma Aldrich	N/A
mCXCR2 BamHI p2a rev 5′ATTCGCGGCCGCGCCACCATGTACCTTGAGGTTAGTG 3′	Sigma Aldrich	N/A
mCXCR3 NotI fwd 5′ATTCGCGGCCGCGCCACCATGTACCTTGAGGTTAGTG 3′	Sigma Aldrich	N/A
mCXCR3 BamHI p2a rev 5′TAATGGATCCCAAGCCCAGGTAGGAGGCCTC 3′	Sigma Aldrich	N/A
mCXCR4 NotI fwd 5′ ATTAGCGGCCGCGCCACCATGGAACCGA TCAGTGTG 3′	Sigma Aldrich	N/A
mCXCR4 BamHI p2a rev 5′GTTCGTGGCTCCGGATCCGCTGGAGTGAAAACTGG 3′	Sigma Aldrich	N/A
mCXCR5 HindIII fwd 5′ATTAAAGCTTGCCACCATGAACTACCCACTAACC 3′	Sigma Aldrich	N/A
mCXCR5 BamHI p2a rev 5′ATTAGGATCCGCTACTTCCCTCACCACCTTCTAG 3′	Sigma Aldrich	N/A
mCXCR6 NotI fwd 5′ATTAGCGGCCGCGCCACCATGGATGATGGGCATCAAG 3′	Sigma Aldrich	N/A
mCXCR6 BamHI p2a rev 5′TAATGGATCCCAATTGGAACATACTGGTGG 3′	Sigma Aldrich	N/A
mCX3CR1 NotI fwd 5′ATTAGCGGCCGCATGTCCACCTCCTTCCCTGAACTG 3′	Sigma Aldrich	N/A
mCX3CR1 BamHI p2a rev 5′ATTAGGATCCTCAGAGCAGGAGAGACCCATC 3′	Sigma Aldrich	N/A
*Recombinant DNA*		
EGFP	MIH Munich	N/A
T-Sapphire	MIH Munich	N/A
CFP	MIH Munich	N/A
EYFP	MIH Munich	N/A
EBFP2	MIH Munich	N/A
mCCR1_P2A_CFP	MIH Munich	N/A
mCCR2_P2A_YFP	MIH Munich	N/A
mCCR3_P2A_YFP	MIH Munich	N/A
mCCR4_P2A_T-Sapphire	MIH Munich	N/A
mCCR5_P2A_GFP	MIH Munich	N/A
mCCR6_P2A_GFP	MIH Munich	N/A
mCCR7_P2A_YFP	MIH Munich	N/A
mCCR8_P2A_YFP	MIH Munich	N/A
mCCR9_P2A_T-Sapphire	MIH Munich	N/A
mCCR10_P2A_BFP	MIH Munich	N/A
mXCR1_p2A_BFP	MIH Munich	N/A
mCXCR1_P2A_T-Sapphire	MIH Munich	N/A
mCXCR2_P2A_T-Sapphire	MIH Munich	N/A
mCXCR3_P2A_T-Sapphire	MIH Munich	N/A
mCXCR4_P2A_BFP	MIH Munich	N/A
mCXCR5_P2A_BFP	MIH Munich	N/A
mCXCR6_P2A_YFP	MIH Munich	N/A
mCX3CR1_P2A_BFP	MIH Munich	N/A
*Software and algorithms*		
FlowJo V10	FlowJo LLC	https://www.flowjo.com
Prism 8	Graphpad	https://www.graphpad.com

## Results

### Generation of a library of chemokine receptors

**Fig. 1:**
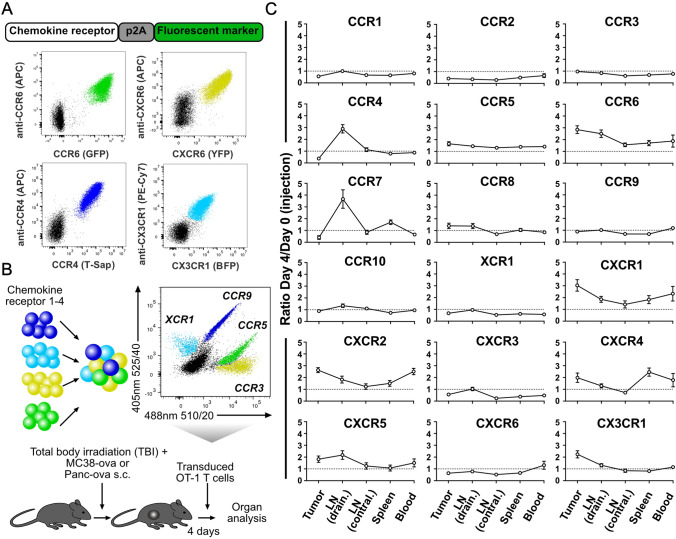
**A** Construct structure and representative flow cytometry plots of co-expressed chemokine receptors and fluorescent proteins. **B** Experimental setup. T cells transduced with different chemokine receptors linked to unique fluorescent proteins are pooled and injected into mice bearing subcutaneous MC38-ova or Panc-ova tumors. The fluorescent proteins GFP, YFP, T-Sapphire and BFP can be discerned by their unique emission pattern after excitation with the 488 nm laser (x-axis, signal shown for a 525/40 nm filter) and 405 nm laser (y-axis, signal shown for a 510/20 nm filter). Example shows a pool of CCR5-GFP, CCR3-YFP, CCR9-T-Sapphire and XCR1-BFP transduced T cells. **C** Homing patterns of T cells expressing engineered murine chemokine receptors in MC38-ova tumor-bearing mice. Day 4/day 0 ratios are calculated by comparing the abundance of T cells, transduced with a given chemokine receptor in the organ infiltrate (day 4 post injection) to their abundance in the cell product before adoptive transfer (day 0). Data in **C** are pooled from 2 to 3 independent experiments

To comprehensively assess the homing profile conveyed to antigen-specific T cells by chemokine receptor engineering, we generated a library of all murine chemokine receptors. Since ligand binding can lead to rapid chemokine receptor internalization and to discern engineered from endogenous chemokine receptor expression, each of these receptors was linked via a P2A sequence to one of four fluorescent proteins: GFP, YFP, T-Sapphire or BFP. In absence of ligand binding, we exemplarily verified that fluorescent protein expression tightly correlated with expression of the respective chemokine receptor (Fig. 1A). Next, we transduced TCR-transgenic OT-1 T cells specific to the SIINFEKL peptide of chicken Ovalbumin (ova) with one chemokine receptor per population. To make assessment of homing patterns more efficient, we used distinct fluorescent tags to assemble up to four populations expressing distinct chemokine receptors and one untransduced population that served as an internal control. This mixed cohort was then transferred into mice bearing subcutaneous MC38-ova (colon carcinoma) or Panc-ova (pancreatic adenocarcinoma) tumors (Fig. 1B). On day 4 post injection, the relative abundance of fluorescently-labeled versus untransduced T cells was measured in tumor, blood, spleen, contralateral and draining lymph nodes and then compared to the relative abundance of fluorescently-labeled versus untransduced T cells in the infusion product (ratio day 4 / day 0; see Methods). Calculating this ratio of day 4 infiltration versus day 0 infusion, revealed that defined chemokine receptors were associated with unique homing patterns. For example, in mice inoculated with either MC38-ova or Panc-ova, CCR7 transduced T cells were overrepresented in lymph nodes, while CXCR4 transduced T cells were overrepresented in spleen (Fig. 1C and Supp. Figure 1).

### Distinct homing patterns of chemokine receptors in vivo

**Fig. 2:**
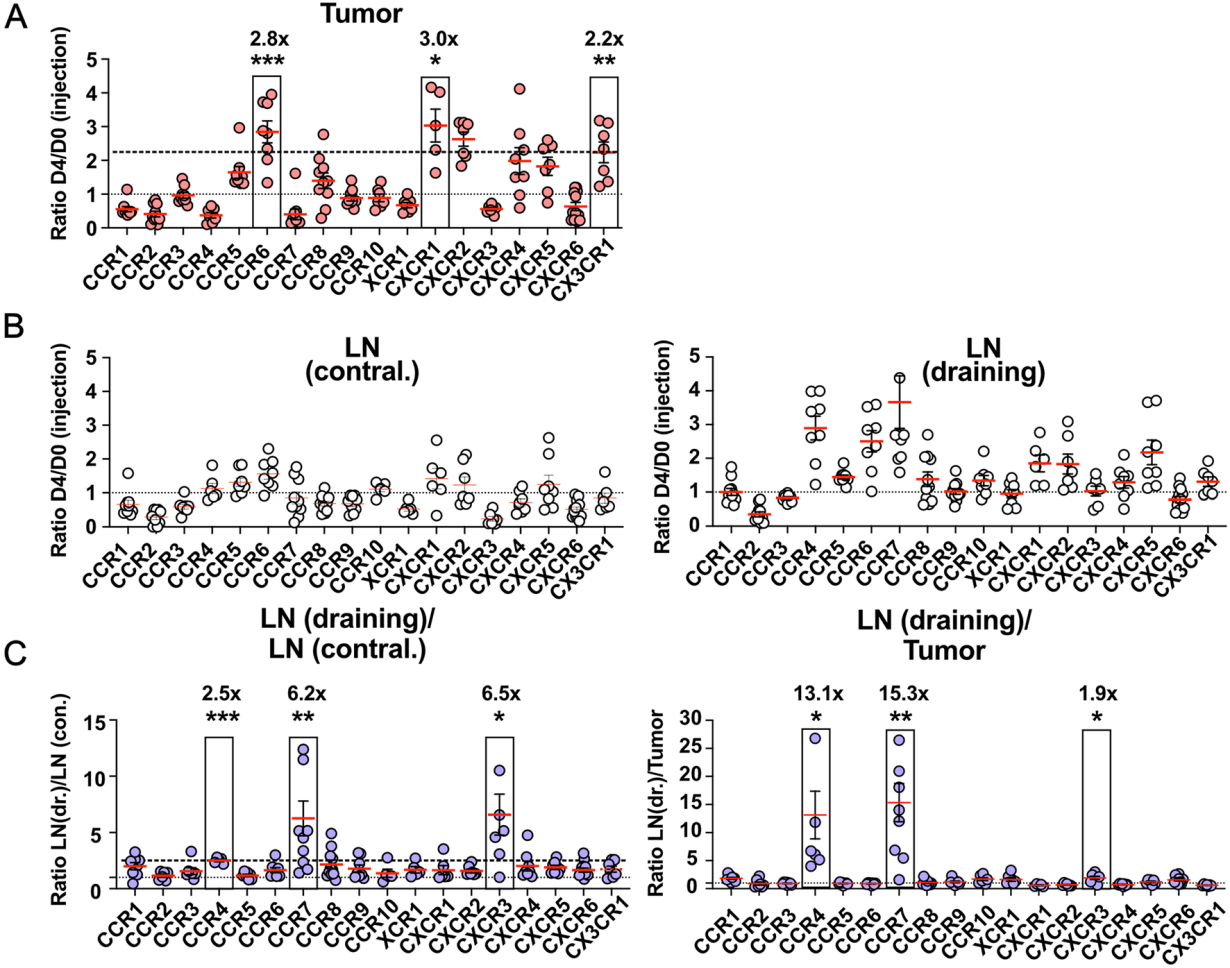
**A** Enrichment ratios of OT-1 T cells engineered with the respective chemokine receptors in MC38-ova tumors on day 4 after injection. Day 4 to day 0 (D4/D0) ratios are calculated relative to chemokine receptor abundance in the infusion product at day 0. **B** Ratios of the abundance of engineered OT-1 T cells in contralateral lymph nodes (left) or tumor-draining lymph nodes (right) on day 4 post injection, compared to the infused product. **C** Relative enrichment ratios of engineered OT-1 T cells. Ratios of D4/D0 enrichment ratios in draining versus contralateral lymph nodes (left) or draining lymph nodes versus tumor (right). Dotted line (fine dots): ratio of 1 (no enrichment). Dotted line (stripes): indicates ratio of lowest highlighted chemokine receptor where applicable. Data are pooled from 2 to 3 independent experiments. Significances in **A** and **C** were calculated as *t*-test against the fixed value 1. * = *p* < 0.05, ** = *p* < 0.01, *** = *p* < 0.001

To identify the best targets for increased tumor homing, we compared the abundance of each chemokine receptor-engineered T cell population in the tumor at day 4 post injection to its abundance in the infusion product (Fig. 2A and Supp. Figure 2A). The top targets for homing into MC38-ova tumors were CCR6, CXCR1, CXCR2 and CX3CR1. Of these, CCR6 and CXCR1 also showed increased infiltration in Panc-ova tumors (Supp. Figure 2A). Since CXCR1 and CXCR2 bind the same targets, we picked only one, namely CXCR1 for further experiments. To answer whether homing into tumor or draining lymph node was more important for anti-tumor function of engineered T cells, we assessed all chemokine receptors regarding their potential to increase immediate homing into lymph nodes (Fig. 2B and Supp. Figure 2B). To particularly select for chemokine receptors that promote homing into the draining lymph nodes and not lymph nodes in general, we furthermore calculated normalized ratios between enrichment into draining lymph nodes and contralateral lymph nodes or tumor, respectively (Fig. 2C). Here, we selected CCR4 and CCR7, which showed the highest ratio of draining to contralateral lymph node enrichment and a particularly high specific enrichment into draining lymph node over the tumor.

### Targeting tumor-specific T cells into tumor-draining lymph nodes or the tumor itself are both successful therapeutic strategies

**Fig. 3:**
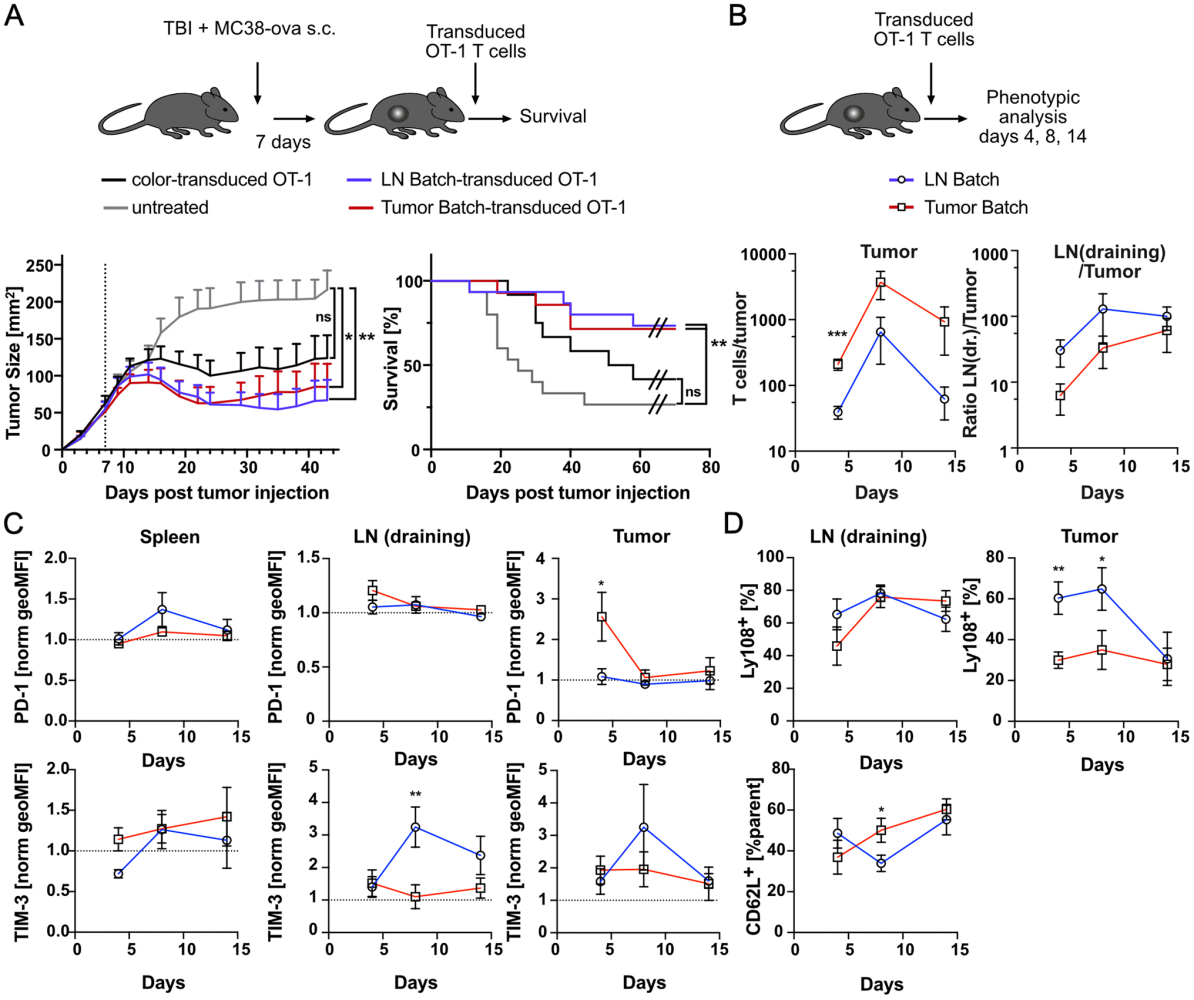
**A** Experimental setup for comparison of treatment efficacy of T cells engineered with lymph node- versus tumor-homing chemokine receptor batches. Tumor growth curves and survival. **B** Experimental setup for measuring differential distribution and phenotype of T cells engineered with lymph node- versus tumor-homing chemokine receptor batches. Kinetic of tumor infiltration and LN (draining)/Tumor ratio for the two batches. **C** Normalized geoMFIs for PD-1 and Tim-3 in spleen, draining lymph nodes and tumor. **D** Expression of CD62L and Slamf6 (Ly108) in draining lymph nodes and Slamf6 in tumor. Data are pooled from 2 different independent experiments. Significances were measured using unpaired *t*-test. * = *p* < 0.05, ** = *p* < 0.01. 400.000 OT-1 were transferred on average in Fig. 3A and B

Next, we used combinatorial transduction to transduce the most promising chemokine receptors in two batches: One batch comprised CCR6, CXCR1 and CX3CR1–all chemokine receptors facilitating homing into MC38-ova tumors. The other batch contained CCR4 and CCR7, which conveyed T cell enrichment in the tumor-draining lymph nodes. In contrast to mice receiving T cells transduced with constructs encoding fluorescent proteins only (color-transduced), both chemokine-receptor-engineered T cell batches showed significant treatment efficacy in relation to untreated mice (Fig. 3A). Next, we analyzed distribution and phenotype of T cells engineered with the two batches at different time points post injection. As expected, the tumor-directed batch led to better T cell accumulation in the tumor at day 4 post injection, whereas the lymph-node homing batch led to relatively more T cell migration to the tumor-draining lymph node (Fig. 3B). However, regarding tumor infiltration, on day 8 post injection the lymph node batch caught up with the tumor batch. Comparing markers for activation and exhaustion, we found that the tumor-homing batch showed higher PD-1 expression early in the tumor, potentially as a sign for immediate antigen encounter (Fig. 3C). The lymph node batch in turn showed higher expression of stemness markers in the draining lymph nodes and Slamf6 in the tumor side (Fig. 3D). Together, it appeared that the tumor-targeting batch preferentially enriched immediately in the tumor, while the lymph node batch showed a better persistence toward the intermediate time point. This longer persistence in the lymph node batch coincided with a relatively higher expression of markers for stemness in the lymph nodes and tumor [24–26].

### Expression of two or more chemokine receptors is not superior over expression of one high-quality receptor

**Fig. 4:**
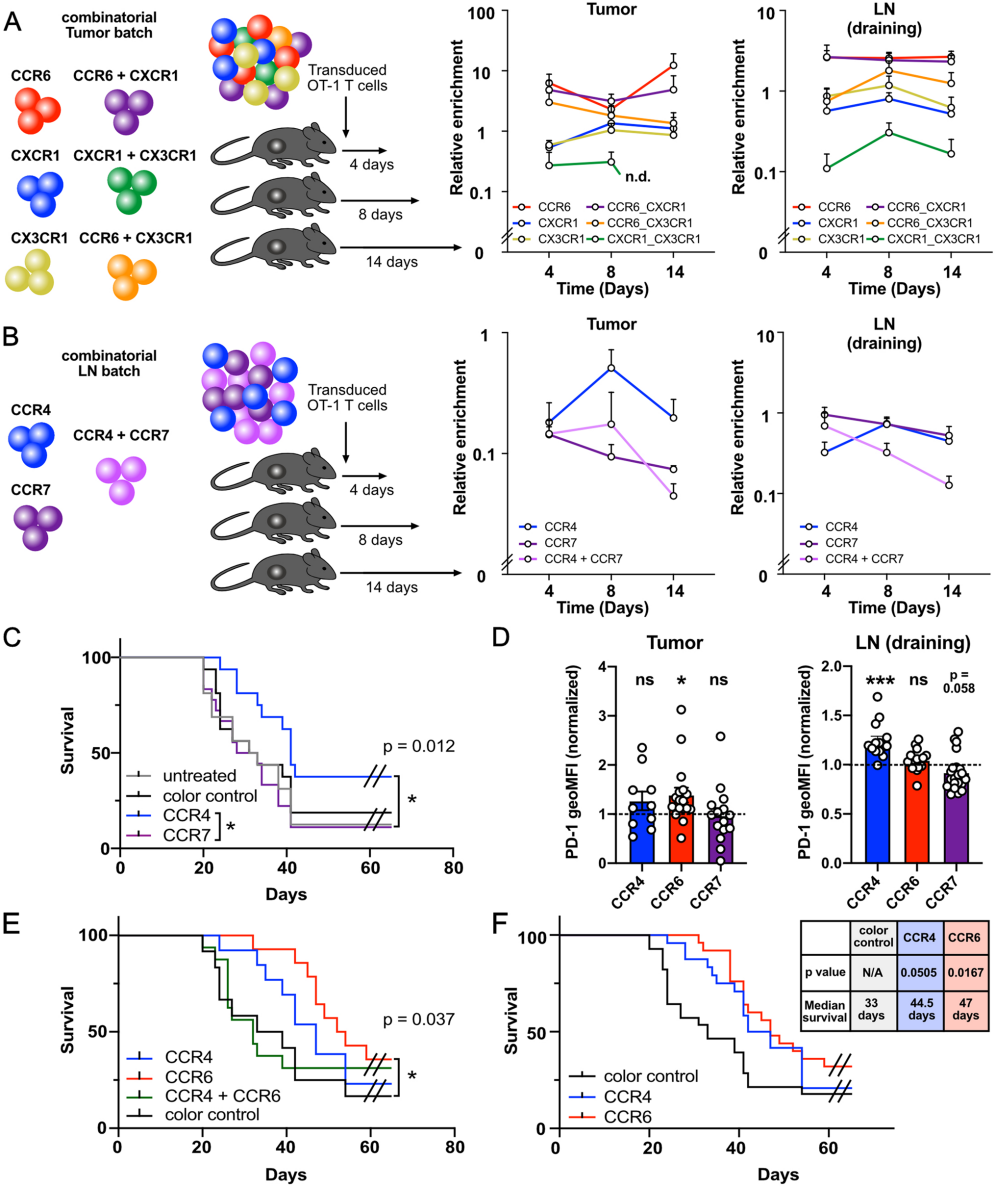
**A** Distribution of T cells expressing individual chemokine receptors or chemokine receptor combinations from the tumor-homing batch in tumor and tumor-draining lymph nodes. **B** Distribution of T cells expressing individual chemokine receptors from the lymph node-homing batch in tumor and tumor-draining lymph node. **C** Survival of MC38-ova tumor-bearing mice treated with CCR4-transduced, CCR7-transduced or control-transduced OT-1 T cells or left untreated. **D** Normalized PD-1 expression of CCR4, CCR6 and CCR7-transduced T cells 4 days post injection in tumor (Left) and draining lymph node (Right). **E** Survival of MC38-ova tumor-bearing mice treated with CCR4-, CCR6-, CCR4/CCR6 double-transduced or control-transduced OT-1 T cells. **F** Survival of MC38-ova tumor-bearing mice treated with CCR4-, CCR6- or control-transduced T cells (all conducted experiments). Data in **A** and **B** are representative of two independent experiments. Data in **C**, **D** and **E** are pooled from two to three independent experiments. Significances for survival experiments in **C**, **E** and **F** were calculated using log-rank test. Significances in **D** were calculated as *t*-test against the fixed value 1. * = *p* < 0.05, ** = *p* < 0.01, *** = *p* < 0.001, 50.000 (4C), 100.000 (4E) and 65.000 (4F) OT-1 T cells were transferred on average

We hypothesized that by expressing more than one receptor that improves homing into the respective target site, migration could be improved through increased signal strength and specificity. To this end, we separated single and multiple transduced OT-1 T cells on days 4, 8 and 14 (Fig. 4A and B). To our surprise, we could not observe a clear synergistic pattern in the tumor batch (Fig. 4A). Instead, CCR6 alone was as efficient or even more efficient in directing T cells into the tumor as was dual transduction with CCR6 and CXCR1 or CCR6 and CX3CR1. Triple-transduced T cells could not be detected in sufficient numbers throughout, although we initially hypothesized that such T cells might outcompete single-transduced T cells over time. For the lymph node batch, the two chemokine receptors individually appeared to have very different homing patterns over time. In the tumor, CCR4 was detectable in numbers that were as low as CCR7 or double transduced T cells on day 4 but then rapidly increased in numbers and persisted better toward day 14. In the draining lymph node, CCR7 transduced T cells were more abundant on day 4 but then slowly decreased over time, whereas CCR4 transduced T cells peaked at day 8, both in lymph node and tumor. The double-transduced T cells mimicked the behavior of one of the singular receptors in each organ: they resembled the pattern of CCR4 in the tumor and CCR7 in the draining lymph node.

### CCR4 and CCR6 enhance adoptive T cell therapy in a non-synergistic manner

Due to their distinct phenotypes, we chose to investigate the therapeutic potential of CCR4 and CCR7 individually (Fig. 4C). While CCR4- transduced T cells showed a therapeutic efficacy, surprisingly, CCR7 did not. For CCR4, we could previously show that exogenous expression facilitates interaction of CD8^+^ T cells with dendritic cells [13]. Since cDC1 express ligands for CCR4 [27], but not CCR7, we hypothesized that the selective treatment advantage of CCR4 vs. CCR7 despite highly similar homing patterns could be attributed to this mechanism. PD-1 is best known as an exhaustion marker, but is also induced by antigen receptor signaling [28]. Therefore, early during immune responses, PD-1 is an activation marker that is upregulated upon effective antigen recognition. We wondered, if T cells transduced with different chemokine receptors differ in their expression of PD-1 early during their anti-tumoral response. Indeed, CCR4 transduced T cells expressed much higher PD-1 levels in the draining lymph nodes, than did T cells transduced with CCR7 on day 4 post injection (Fig. 4D). This suggested that indeed CCR4 enhanced, whereas CCR7 potentially even impaired antigen recognition in draining lymph nodes, which might explain why the treatment efficacy of the lymph node batch appeared to be largely conferred by CCR4. Lastly, because CCR4 and CCR6 both conferred increased therapeutic efficacies, we tested whether co-transduction of these two chemokine receptors improves anti-tumor efficacy (Fig. 4E). However, we could not detect a synergistic effect. Single-chemokine receptor transduced T cells with CCR4 or CCR6 both led to longer median survival than CCR4/CCR6 double-transduced T cells. Since CCR6 transduced T cells showed increased PD-1 expression in the tumor and CCR4 transduced T cells in draining lymph nodes (Fig. 4D), one possible reason why this combination approach did not work was that CCR4/CCR6 double-transduced T cells become exhausted by additive PD-1 upregulation via CCR4 and CCR6. Indeed, when analyzing PD-1 expression in draining lymph nodes late after infusion, we found CCR4/CCR6 double-transduced T cells to express significantly higher levels of PD-1 than CCR6 transduced T cells (Supp. Figure 3B). Combining data from all conducted experiments, CCR6-transduced T cells significantly and CCR4-transduced T cells almost significantly (*p* = 0.0505) prolonged survival compared to control-transduced T cells (median survival of 47 and 44.5 vs. 33 days, respectively). Thus, it appears that both tumor- and tumor-draining lymph node targeting of T cells can improve therapeutic efficacy of adoptive T cell therapy. However, at least for lymph node targeting, it appears that additional factors beyond simple organ enrichment must be considered in order to identify a therapeutically active chemokine receptor.

## Discussion

Lack of migration of effector immune cells into solid tumors is a crucial bottleneck for adoptive T cell therapy. Engineering T cells with chemokine receptors can overcome this limitation. However, so far, no successful strategy using chemokine receptor transduced T cells has been approved for therapy in humans.

In this study, we characterized different homing patterns for each chemokine receptor. Over time the organ distribution of engineered T cells will be influenced by factors other than homing, such as proliferation. Therefore, we assessed homing patterns at day 4 post injection, an early time point at which we believe organ distribution will largely be influenced by homing. We identified chemokine receptors that improved homing into tumor or tumor-draining lymph nodes. So far, pre-clinical studies have largely focused on chemokine receptors that increase homing directly into the tumor. However, one could imagine that migration into tumor-draining lymph nodes, the side with most cells with a stem-cell like phenotype [20], could confer better anti-tumor efficacy. In our screen, we found two chemokine receptors to very specifically direct T cells into tumor-draining lymph nodes: CCR4 and CCR7.

At first sight, it is surprising that CCR7 expression leads to preferential recruitment to the draining lymph node, as the ligands CCL19 and CCL21 are thought to show homeostatic expression in all lymph nodes [29]. CD8^+^ T cells endogenously express chemokine receptors that lead to homing into activated lymph nodes such as CXCR3 [14, 30]. Thus, we suspect that expression of CCR7 might increase the dwell time in lymph nodes in general, but additional, endogenously expressed receptors lead to preferential accumulation in tumor-draining lymph nodes.

Despite remarkably similar homing patterns, only CCR4-transduced T cells were responsible for the observed treatment effect of the lymph node batch. We hypothesized that it might be crucial which cell type expresses the attracting chemokine in the lymph node. CCR4 ligands are predominantly expressed by dendritic cells [27], while CCR7 ligands are expressed by stromal cells [31]. Fitting this hypothesis, CCR4 and CCR7 transduced T cells showed clearly distinct expression of PD-1 on day 4 post injection. This early after injection, high PD-1 expression is unlikely to be a sign of exhaustion, but rather suggests recent antigen receptor signaling [32]. While CCR4 showed the most significant upregulation of PD-1 of all tested chemokine receptors in draining lymph nodes, CCR7 showed lower than average expression of PD-1. In our screen, all chemokine receptors showing increased PD-1 expression on day 4 post infection either match chemokines secreted by dendritic cells (CCR5, CXCR3) [30, 33] or respond to chemokines that also attract dendritic cells (XCR1). The ligands for CCR7, however, are predominantly expressed by fibroblastic reticular cells in lymph nodes [31]. Thus, once in the lymph node, CCL19 and CCL21 might, in fact, lead T cells away from antigen-presenting cells, while CCR4 enhances interaction with dendritic cells in part via increasing the affinity of LFA-1 [13].

Concerning the tumor-homing batch, we asked whether transduction with multiple chemokine receptors that direct T cells into the tumor might increase functionality. However, we could not observe such a synergistic effect. Instead, overexpression of CCR6 in the MC38-ova model was sufficient and even superior over T cells co-transduced with other tumor-homing receptors. This might be due to various reasons. Potentially, transduction with multiple receptors decreases fitness of the transduced T cells. In this case, generating strategies that rely on expression of receptors via alternative delivery methods, such as CRISPR/Cas9 editing might improve efficacy [34, 35]. Alternatively, co-expression of two or more chemokine receptors could lead to immediate immigration at the expense of maintaining a pool of stem-like cells in the secondary lymphoid organs, which could lead to decreased persistence over time.

Last, we assessed whether a combination of the best tumor- and lymph node-homing chemokine receptors might show a synergistic effect. However, co-transduction of CCR4 and CCR6 was not superior over single transductions, again suggesting that engineering of T cells with one high-quality receptor provides better efficacy than co-transductions. An additional reason for decreased functionality of multiple-transduced cells may be a synergistic PD-1 induction dependent on different chemokine receptors: CCR6 showed increased PD-1 expression in the tumor and CCR4 in draining lymph nodes. We found CCR4/CCR6 double-transduced T cells to express higher levels of PD-1 than CCR6-transduced T cells in endpoint analyses, suggesting that double-transduced T cells might have an increased risk of undergoing T cell exhaustion.

Our study is the first to assess the potential of all murine chemokine receptors to influence homing of CD8^+^ T cells in tumor-bearing mice. Our data indicate that equipment of T cells with one high-quality chemokine receptor is sufficient for enhancing the therapeutic potential of adoptively transferred T cells into solid tumors. Transduction with CCR6, a chemokine receptor known to recruit immunosuppressive myeloid cells and Treg into the tumor [36], showed the best efficacy. The efficacy of CCR6-transduced T cells for immunotherapy has only been studied in xenograft models, where the tumor xenograft is the only source of CCR6 ligands [37, 38]. Furthermore, we could show that directing T cells into the tumor-draining lymph node via CCR4 can enhance therapeutic efficacy. However, the success of directing tumor-reactive T cells into draining lymph nodes likely depended on the cell type releasing the attracting chemokine, as CCR7-transduced T cells showed similar homing but no therapeutic effect.

## Supplementary Information

Below is the link to the electronic supplementary material.

Supplementary file1 (PDF 749 KB)

Supplementary file1 (PDF 1358 KB)

Supplementary file1 (PDF 700 KB)

## References

[1] Curiel TJ, Coukos G, Zou L, Alvarez X, Cheng P, Mottram P, Evdemon-Hogan M, Conejo-Garcia JR, Zhang L, Burow M, Zhu Y, Wei S, Kryczek I, Daniel B, Gordon A, Myers L, Lackner A, Disis ML, Knutson KL, Chen L, Zou W (2004). Specific recruitment of regulatory T cells in ovarian carcinoma fosters immune privilege and predicts reduced survival. Nat Med.

[2] Facciabene A, Peng X, Hagemann IS, Balint K, Barchetti A, Wang LP, Gimotty PA, Gilks CB, Lal P, Zhang L, Coukos G (2011). Tumour hypoxia promotes tolerance and angiogenesis via CCL28 and T(reg) cells. Nature.

[3] Chang AL, Miska J, Wainwright DA, Dey M, Rivetta CV, Yu D, Kanojia D, Pituch KC, Qiao J, Pytel P, Han Y, Wu M, Zhang L, Horbinski CM, Ahmed AU, Lesniak MS (2016). CCL2 produced by the glioma microenvironment is essential for the recruitment of regulatory T cells and myeloid-derived suppressor cells. Cancer Res.

[4] Schlecker E, Stojanovic A, Eisen C, Quack C, Falk CS, Umansky V, Cerwenka A (2012). Tumor-infiltrating monocytic myeloid-derived suppressor cells mediate CCR5-dependent recruitment of regulatory T cells favoring tumor growth. J Immunol.

[5] Gu H, Deng W, Zheng Z, Wu K, Sun F (2021). CCL2 produced by pancreatic ductal adenocarcinoma is essential for the accumulation and activation of monocytic myeloid-derived suppressor cells. Immun Inflamm Dis.

[6] Fujita M, Zhu X, Ueda R, Sasaki K, Kohanbash G, Kastenhuber ER, McDonald HA, Gibson GA, Watkins SC, Muthuswamy R (2009). Effective immunotherapy against murine gliomas using type 1 polarizing dendritic cells—significant roles of CXCL10. Can Res.

[7] Harlin H, Meng Y, Peterson AC, Zha Y, Tretiakova M, Slingluff C, McKee M, Gajewski TF (2009). Chemokine expression in melanoma metastases associated with CD8+ T-cell recruitment. Can Res.

[8] Chow MT, Ozga AJ, Servis RL, Frederick DT, Lo JA, Fisher DE, Freeman GJ, Boland GM, Luster AD (2019). Intratumoral activity of the CXCR3 chemokine system is required for the efficacy of Anti-PD-1 therapy. Immunity.

[9] Moon EK, Carpenito C, Sun J, Wang L-CS, Kapoor V, Predina J, Powell DJ, Riley JL, June CH, Albelda SM (2011). Expression of a functional CCR2 receptor enhances tumor localization and tumor eradication by retargeted human T cells expressing a mesothelin-specific chimeric antibody receptor. Clin Cancer Res.

[10] Lesch S, Blumenberg V, Stoiber S, Gottschlich A, Ogonek J, Cadilha BL, Dantes Z, Rataj F, Dorman K, Lutz J, Karches CH, Heise C, Kurzay M, Larimer BM, Grassmann S, Rapp M, Nottebrock A, Kruger S, Tokarew N, Metzger P, Hoerth C, Benmebarek M-R, Dhoqina D, Grünmeier R, Seifert M, Oener A, Umut Ö, Joaquina S, Vimeux L, Tran T, Hank T, Baba T, Huynh D, Megens RTA, Janssen K-P, Jastroch M, Lamp D, Ruehland S, Di Pilato M, Pruessmann JN, Thomas M, Marr C, Ormanns S, Reischer A, Hristov M, Tartour E, Donnadieu E, Rothenfusser S, Duewell P, König LM, Schnurr M, Subklewe M, Liss AS, Halama N, Reichert M, Mempel TR, Endres S, Kobold S (2021). T cells armed with C-X-C chemokine receptor type 6 enhance adoptive cell therapy for pancreatic tumours. Nat Biomed Eng.

[11] Siddiqui I, Erreni M, van Brakel M, Debets R, Allavena P (2016). Enhanced recruitment of genetically modified CX3CR1-positive human T cells into Fractalkine/CX3CL1 expressing tumors: importance of the chemokine gradient. J Immunother Cancer.

[12] Peng W, Ye Y, Rabinovich BA, Liu C, Lou Y, Zhang M, Whittington M, Yang Y, Overwijk WW, Lizée G (2010). Transduction of tumor-specific T cells with CXCR2 chemokine receptor improves migration to tumor and antitumor immune responses. Clin Cancer Res.

[13] Rapp M, Grassmann S, Chaloupka M, Layritz P, Kruger S, Ormanns S, Rataj F, Janssen KP, Endres S, Anz D, Kobold S (2016). C-C chemokine receptor type-4 transduction of T cells enhances interaction with dendritic cells, tumor infiltration and therapeutic efficacy of adoptive T cell transfer. Oncoimmunology.

[14] Nagarsheth N, Wicha MS, Zou W (2017). Chemokines in the cancer microenvironment and their relevance in cancer immunotherapy. Nat Rev Immunol.

[15] Fang WB, Jokar I, Zou A, Lambert D, Dendukuri P, Cheng N (2012). CCL2/CCR2 chemokine signaling coordinates survival and motility of breast cancer cells through Smad3 protein-and p42/44 mitogen-activated protein kinase (MAPK)-dependent mechanisms. J Biol Chem.

[16] Wente MN, Gaida MM, Mayer C, Michalski CW, Haag N, Giese T, Felix K, Bergmann F, Giese NA, Friess H (2008). Expression and potential function of the CXC chemokine CXCL16 in pancreatic ductal adenocarcinoma. Int J Oncol.

[17] Li A, Dubey S, Varney ML, Dave BJ, Singh RK (2003). IL-8 directly enhanced endothelial cell survival, proliferation, and matrix metalloproteinases production and regulated angiogenesis. J Immunol.

[18] Bonapace L, Coissieux MM, Wyckoff J, Mertz KD, Varga Z, Junt T, Bentires-Alj M (2014). Cessation of CCL2 inhibition accelerates breast cancer metastasis by promoting angiogenesis. Nature.

[19] Anz D, Rapp M, Eiber S, Koelzer VH, Thaler R, Haubner S, Knott M, Nagel S, Golic M, Wiedemann GM, Bauernfeind F, Wurzenberger C, Hornung V, Scholz C, Mayr D, Rothenfusser S, Endres S, Bourquin C (2015). Suppression of intratumoral CCL22 by type i interferon inhibits migration of regulatory T cells and blocks cancer progression. Cancer Res.

[20] Connolly KA, Kuchroo M, Venkat A, Khatun A, Wang J, William I, Hornick NI, Fitzgerald BL, Damo M, Kasmani MY, Cui C, Fagerberg E, Monroy I, Hutchins A, Cheung JF, Foster GG, Mariuzza DL, Nader M, Zhao H, Cui W, Krishnaswamy S, Joshi NS (2021). A reservoir of stem-like CD8(+) T cells in the tumor-draining lymph node preserves the ongoing antitumor immune response. Sci Immunol.

[21] Flommersfeld S, Böttcher JP, Ersching J, Flossdorf M, Meiser P, Pachmayr LO, Leube J, Hensel I, Jarosch S, Zhang Q, Chaudhry MZ, Andrae I, Schiemann M, Busch DH, Cicin-Sain L, Sun JC, Gasteiger G, Victora GD, Höfer T, Buchholz VR, Grassmann S (2021). Fate mapping of single NK cells identifies a type 1 innate lymphoid-like lineage that bridges innate and adaptive recognition of viral infection. Immunity.

[22] Grassmann S, Mihatsch L, Mir J, Kazeroonian A, Rahimi R, Flommersfeld S, Schober K, Hensel I, Leube J, Pachmayr LO, Kretschmer L, Zhang Q, Jolly A, Chaudhry MZ, Schiemann M, Cicin-Sain L, Höfer T, Busch DH, Flossdorf M, Buchholz VR (2020). Early emergence of T central memory precursors programs clonal dominance during chronic viral infection. Nat Immunol.

[23] Grassmann S, Pachmayr LO, Leube J, Mihatsch L, Andrae I, Flommersfeld S, Oduro J, Cicin-Sain L, Schiemann M, Flossdorf M, Buchholz VR (2019). Distinct surface expression of activating receptor Ly49H drives differential expansion of NK cell clones upon murine cytomegalovirus infection. Immunity.

[24] Tsui C, Kretschmer L, Rapelius S, Gabriel SS, Chisanga D, Knöpper K, Utzschneider DT, Nüssing S, Liao Y, Mason T, Torres SV, Wilcox SA, Kanev K, Jarosch S, Leube J, Nutt SL, Zehn D, Parish IA, Kastenmüller W, Shi W, Buchholz VR, Kallies A (2022). MYB orchestrates T cell exhaustion and response to checkpoint inhibition. Nature.

[25] Utzschneider DT, Gabriel SS, Chisanga D, Gloury R, Gubser PM, Vasanthakumar A, Shi W, Kallies A (2020). Early precursor T cells establish and propagate T cell exhaustion in chronic infection. Nat Immunol.

[26] Im SJ, Hashimoto M, Gerner MY, Lee J, Kissick HT, Burger MC, Shan Q, Hale JS, Lee J, Nasti TH, Sharpe AH, Freeman GJ, Germain RN, Nakaya HI, Xue H-H, Ahmed R (2016). Defining CD8+ T cells that provide the proliferative burst after PD-1 therapy. Nature.

[27] Rapp M, Wintergerst MWM, Kunz WG, Vetter VK, Knott MML, Lisowski D, Haubner S, Moder S, Thaler R, Eiber S, Meyer B, Rohrle N, Piseddu I, Grassmann S, Layritz P, Kuhnemuth B, Stutte S, Bourquin C, von Andrian UH, Endres S, Anz D (2019). CCL22 controls immunity by promoting regulatory T cell communication with dendritic cells in lymph nodes. J Exp Med.

[28] Lu P, Youngblood BA, Austin JW, Mohammed AU, Butler R, Ahmed R, Boss JM (2014). Blimp-1 represses CD8 T cell expression of PD-1 using a feed-forward transcriptional circuit during acute viral infection. J Exp Med.

[29] Chen K, Bao Z, Tang P, Gong W, Yoshimura T, Wang JM (2018). Chemokines in homeostasis and diseases. Cell Mol Immunol.

[30] Groom JR, Richmond J, Murooka TT, Sorensen EW, Sung JH, Bankert K, von Andrian UH, Moon JJ, Mempel TR, Luster AD (2012). CXCR3 chemokine receptor-ligand interactions in the lymph node optimize CD4+ T helper 1 cell differentiation. Immunity.

[31] Link A, Vogt TK, Favre S, Britschgi MR, Acha-Orbea H, Hinz B, Cyster JG, Luther SA (2007). Fibroblastic reticular cells in lymph nodes regulate the homeostasis of naive T cells. Nat Immunol.

[32] Chikuma S, Terawaki S, Hayashi T, Nabeshima R, Yoshida T, Shibayama S, Okazaki T, Honjo T (2009). PD-1-mediated suppression of IL-2 production induces CD8+ T cell anergy in vivo. J Immunol.

[33] Rawat K, Tewari A, Li X, Mara AB, King WT, Gibbings SL, Nnam CF, Kolling FW, Lambrecht BN, Jakubzick CV (2023). CCL5-producing migratory dendritic cells guide CCR5+ monocytes into the draining lymph nodes. J Exp Med.

[34] Eyquem J, Mansilla-Soto J, Giavridis T, van der Stegen SJ, Hamieh M, Cunanan KM, Odak A, Gönen M, Sadelain M (2017). Targeting a CAR to the TRAC locus with CRISPR/Cas9 enhances tumour rejection. Nature.

[35] Schober K, Müller TR, Gökmen F, Grassmann S, Effenberger M, Poltorak M, Stemberger C, Schumann K, Roth TL, Marson A, Busch DH (2019). Orthotopic replacement of T-cell receptor α- and β-chains with preservation of near-physiological T-cell function. Nat Biomed Eng.

[36] Kadomoto S, Izumi K, Mizokami A (2020). The CCL20-CCR6 axis in cancer progression. Int J Mol Sci.

[37] Wang J, Wang Y, Pan H, Zhao L, Yang X, Liang Z, Shen X, Zhang J, Yang J, Zhu Y, Xun J, Liang Y, Lin Q, Liang H, Li M, Zhu H (2023). Chemokine receptors CCR6 and PD1 blocking scFv E27 enhances anti-EGFR CAR-T therapeutic efficacy in a preclinical model of human non-small cell lung carcinoma. Int J Mol Sci.

[38] Jin L, Cao L, Zhu Y, Cao J, Li X, Zhou J, Liu B, Zhao T (2021). Enhance anti-lung tumor efficacy of chimeric antigen receptor-T cells by ectopic expression of C-C motif chemokine receptor 6. Sci Bull.

