# Supplementary figures and images for: Unbiased chemokine receptor screening reveals similar efficacy of lymph node- and tumor-targeted T cell immunotherapy
Source: Cancer Immunol Immunother. 2023 Jun 10;72(9):3111–24. doi: 10.1007/s00262-023-03472-w (PMC10412482; doi:10.1007/s00262-023-03472-w)

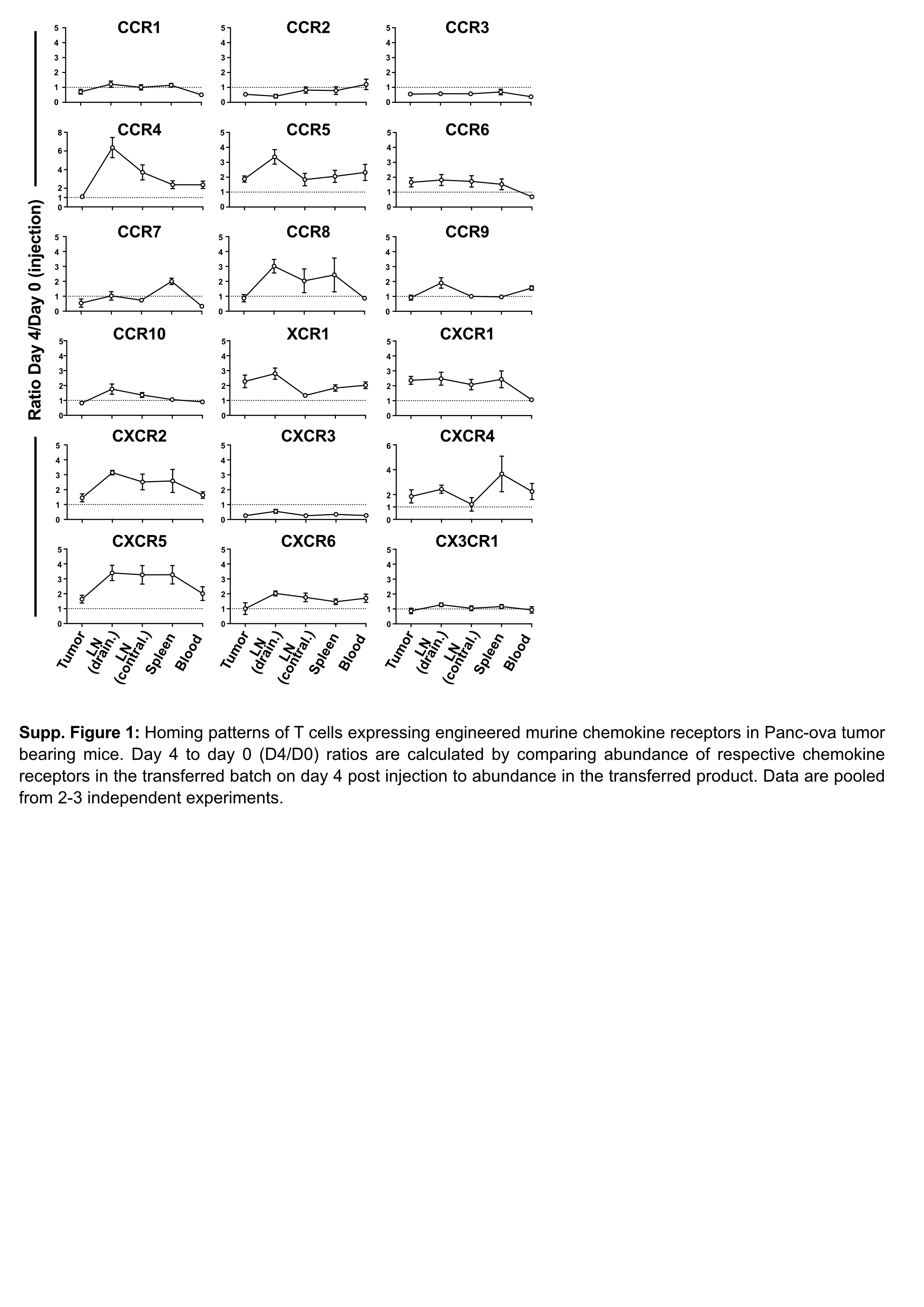

Supplement: Supplementary file 1 — Supplementary file1 (PDF 749 KB) [file 262_2023_3472_MOESM1_ESM.jpg]

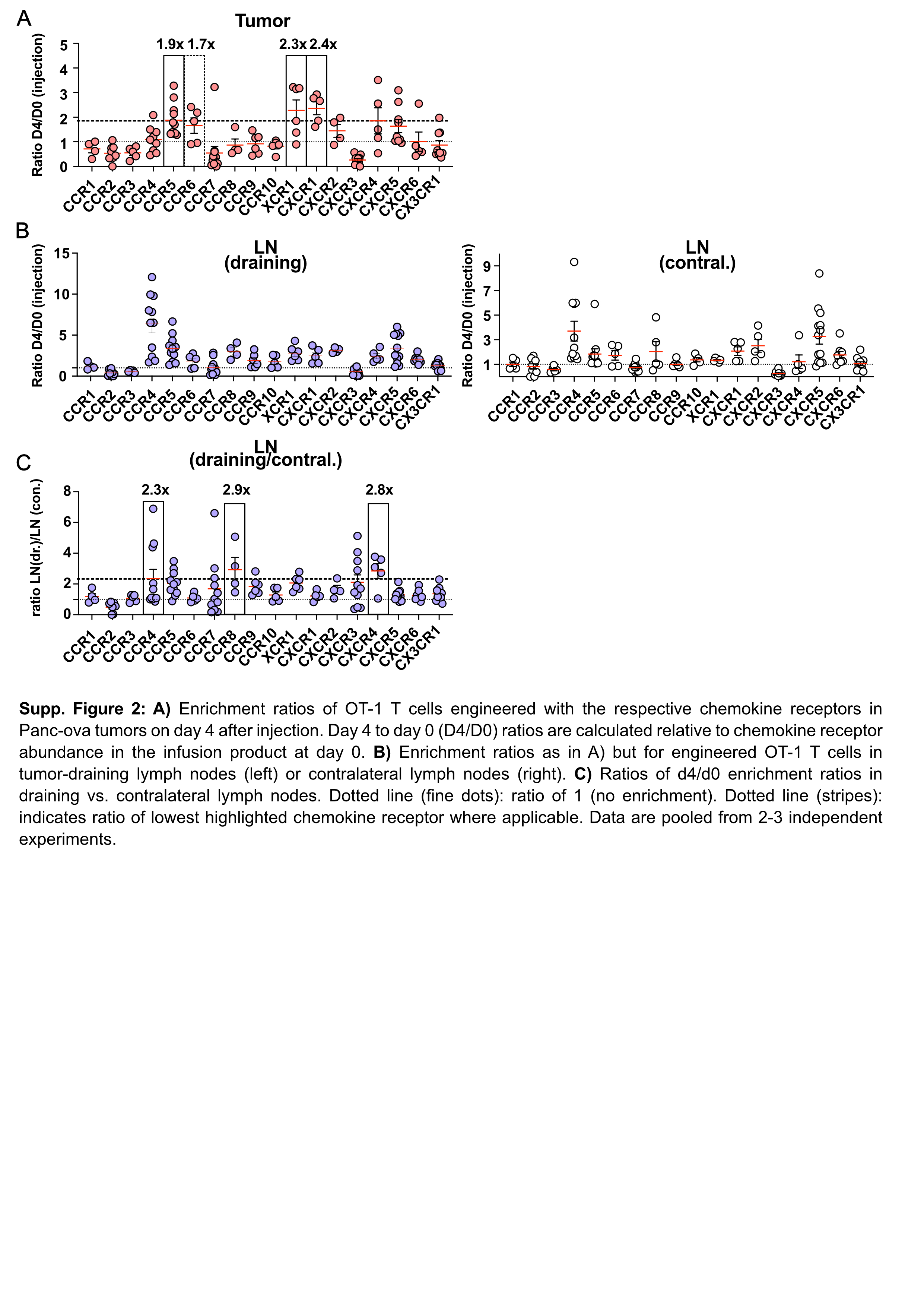

Supplement: Supplementary file 2 — Supplementary file1 (PDF 1358 KB) [file 262_2023_3472_MOESM2_ESM.jpg]

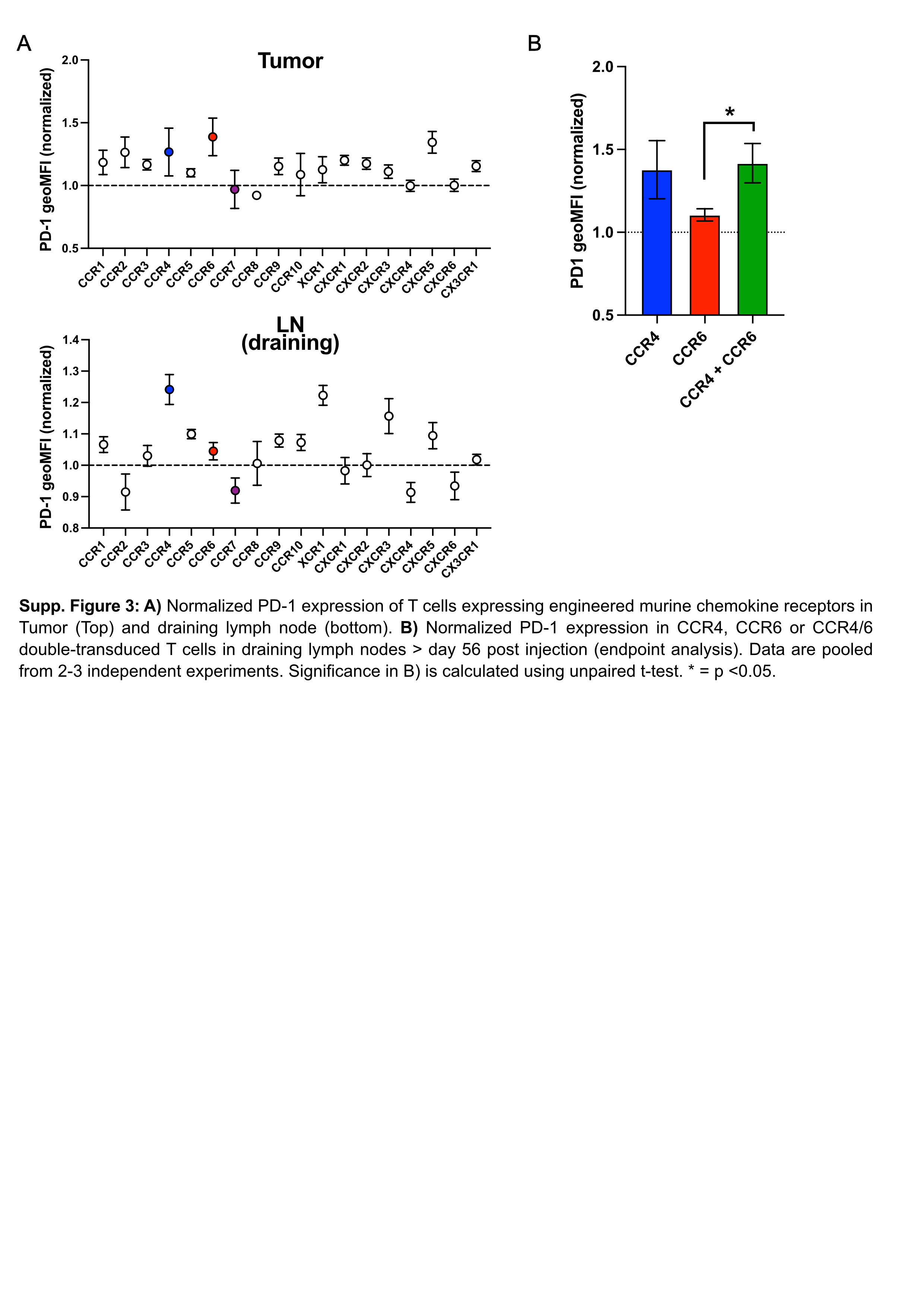

Supplement: Supplementary file 3 — Supplementary file1 (PDF 700 KB) [file 262_2023_3472_MOESM3_ESM.jpg]
